# A Custom-Developed Device for Testing Tensile Strength and Elasticity of Vascular and Intestinal Tissue Samples for Anastomosis Regeneration Research

**DOI:** 10.3390/s24185984

**Published:** 2024-09-15

**Authors:** Zoltan Attila Godo, Laszlo Adam Fazekas, Gergo Fritsch, Balazs Szabo, Norbert Nemeth

**Affiliations:** 1 Department of Information Technology, Faculty of Informatics, University of Debrecen, Kassai Str. 26, H-4028 Debrecen, Hungary; godo.zoltan@inf.unideb.hu (Z.A.G.); fritschgergo@mailbox.unideb.hu (G.F.); 2 Department of Operative Techniques and Surgical Research, Faculty of Medicine, University of Debrecen, Moricz Zsigmond Str. 22, H-4032 Debrecen, Hungary; fazekas.laszlo@med.unideb.hu (L.A.F.); balazs.szabo@med.unideb.hu (B.S.)

**Keywords:** tensile strength, elasticity, vascular anastomosis, intestinal anastomosis, experimental model

## Abstract

Optimizing the regeneration process of surgically created anastomoses (blood vessels, intestines, nerves) is an important topic in surgical research. One of the most interesting parameter groups is related to the biomechanical properties of the anastomoses. Depending on the regeneration process and its influencing factors, tensile strength and other biomechanical features may change during the healing process. Related to the optimal specimen size, the range and accuracy of measurements, and applicability, we have developed a custom-tailored microcontroller-based device. In this paper, we describe the hardware and software configuration of the latest version of the device, including experiences and comparative measurements of tensile strength and elasticity of artificial materials and biopreparate tissue samples. The machine we developed was made up of easily obtainable parts and can be easily reproduced on a low budget. The basic device can apply a force of up to 40 newtons, and can grasp a 0.05–1 cm wide, 0.05–1 cm thick tissue. The length of the test piece on the rail should be between 0.3 and 5 cm. Low production cost, ease of use, and detailed data recording make it a useful tool for experimental surgical research.

## 1. Introduction

Performing various anastomoses is an important part of numerous surgical interventions on the vasculature as well as on the gastrointestinal tract. Independently of the anastomoses’ geometry (end-to-end, end-to-side, side-to-side), it is crucial to provide surgical safety and the optimal condition for the healing process as regeneration or maturation of the anastomoses [1,2,3,4,5,6,7,8,9,10,11]. It is an old but always actual topic in surgical research, since numerous factors may influence the anastomoses’ regeneration [1,2,3,5,6,12,13,14,15].

Proper healing process and biomechanical properties are key issues for anastomosis safety [16,17,18,19,20,21,22]. Numerous experimental and clinical studies deal with this issue [23,24,25,26,27,28,29,30,31]. Concerning the objectively measurable parameters, besides functional variables, decisive factors are the histological and biomechanical properties [16,17,18,19,32]. In experimental conditions, properly measuring the tensile strengths of vascular anastomoses or intestinal anastomoses also has great importance. However, limited technological possibilities are available especially related to the optimal specimen size, adjustability, the tissue-fixing parts, and the clinically relevant parameters provided by a device.

Previously, we developed a cost-effective custom-made device to accurately test tensile strengths and elastic properties of various specimens, focusing on vascular, nerve, and intestinal anastomoses regeneration studies [33]. To improve its accuracy, mechanics, stability, and widen its application possibilities, and to optimize the data recording and data analyzing features, we aimed to develop a new version.

In this paper, our aim was to describe the methodological details of a tensile strength measuring device developed for tissue samples tested in anastomosis regeneration studies, showing technical details, repeatability, accuracy, and some initial experiences on various tissue specimens.

## 2. Materials and Methods—Description of the Device

### 2.1. The Hardware

#### 2.1.1. The Skeleton

**Figure 1:**
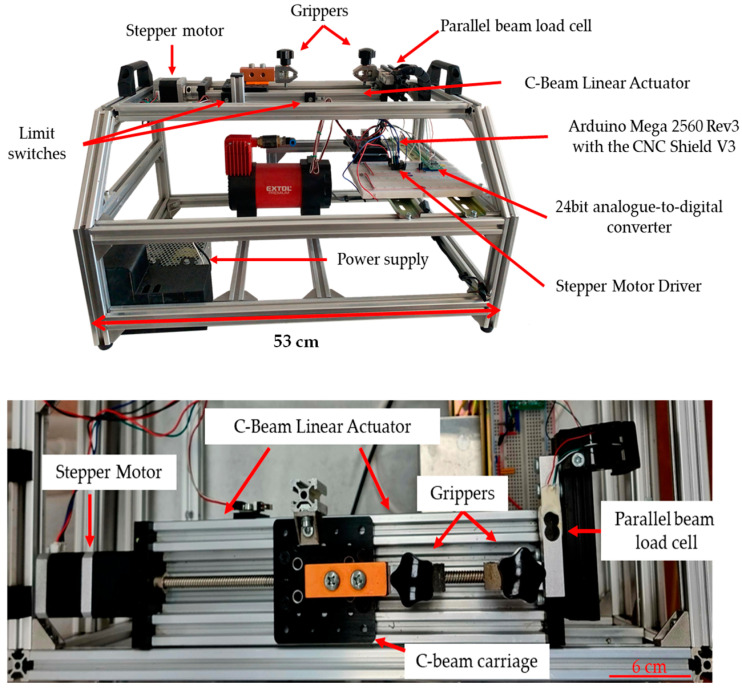
Representative picture of the structure of the device from different views. A robust frame is required for stable and accurate operation of the instrument.

An important aspect of the design of the device was to build it on a stable and strong frame. It may not seem like an important consideration, but it is essential. As it will stretch and tear during use, it will be subjected to considerable forces from the motor. Precise data acquisition sensors can only measure accurate values if the device does not move or vibrate significantly during use, as opposed to the previous machine [33], which was essentially a metal box. That allowed a much greater torsion of the body during testing, which subsequently resulted in a less precise measurement. In addition to a robust construction, the aim was to have a lightweight design. It should be easy to move between the laboratory, the experimental operating theatre, or the classroom. Therefore, the frame was built from high-tech aluminum profiles (Figure 1).

#### 2.1.2. Tension Mechanics

Accurate mechanics is a fundamental requirement for exact measurements. The traction unit is based on an HPV7 C-Beam Linear Actuator (Guangzhou Hanpose 3D Technology Co., Ltd., Guangzhou, China). The actuator is connected to a C-shaped aluminum machine-building element. The pulling mechanism is driven by a NEMA17 stepper motor (42BYGHW811M, Kuongshun Electronic Ltd., Shenzhen, China), which is guided by a Stepper Motor Driver Carrier (DRV882, Pololu Robotics and Electronics, Las Vegas, NV, USA) applied on an Arduino CNC Shield V3 (Kuongshun Electronic Ltd., Shenzhen, China). A T8 trapezoidal spindle is connected to this stepper motor via a clutch. This mechanism moves a C-beam carriage via a splinted trapezoidal nut. In the case of the old device, one of the grippers was pulled by a plastic belt, which was connected to the axes of the stepper motor. This meant that the pulling force and the pulling distances were non-linear. As the motor wound up the belt, the diameter of the rolled-up belt became larger; therefore, each step by the motor was bigger, and the applied force was larger. Also, the belt was flexible, so the transfer of the pulling force was less efficient, making the measurement less constant.

The machine built prior to that had a much weaker clamp, which could not grab anything thicker than 2 mm.

**Figure 2:**
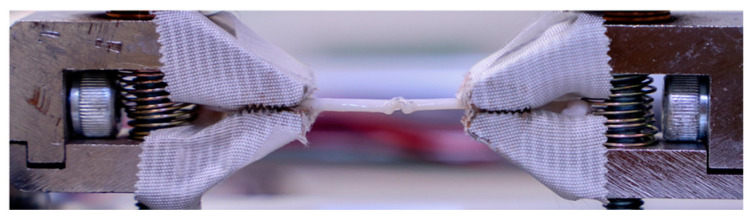
Representative picture of the HJJ-001 type grippers while testing a chicken sciatic nerve (biopreparate).

As a first attempt, we used a 3D printer to make jaws hold different tissues, such as blood vessels. This was not suitable because they were not stable enough due to the high pulling force. In the case of the new device, we improved it further and then used HJJ-001 type grippers (Baoshishan Co., Dalian, China) developed to industry standards for direct pulling purposes. These clamps are certified up to 500 N pull force. The surface of the gripper is ribbed to ensure a more stable grip without tearing or cutting the sample (Figure 2).

#### 2.1.3. Force Sensors

A force measurement system is installed to measure the load applied to the vessel by the pulling mechanism. The measuring system consists of a load cell with a strain gauge stamp (TAL220, Deecom Technology, Kuala Lumpur, Malaysia) and an analog-to-digital converter for the digitalization of the analog data measured on the load cell.

The strain gauge stamp converts mechanical changes in the fixed body into electrical resistance changes. As the body deforms, the strain gauge stamp attached to it undergoes an elastic deformation, while the resistance of the conductors inside it changes. The type we have chosen is a widely used load cell. It is popularly used in testing apparatus, measuring tools, compression, and tension equipment. It contains 2 strain gauges with a measuring capacity of 5 kg (49.03 N). Further specifications of parallel beam load cell (aluminum-alloy): 120%FS safe overload, 150%FS ultimate overload, 1.0 ± 0.15 mV/V rated output, 5~10 Vdc excitation voltage, ±0.05%FS combined error, ±0.05%FS hysteresis, ±0.03%FS repeatability, ±0.05%FS/3 min creep, 1000 ± 15 Ω input and 1000 ± 10 Ω output resistance, operating temperature range: −10 to +55 °C, and temperature coefficient: ±0.05%FS/10 °C. The compensated temperature range is certified from −10 to +40 °C. The instrument was used at constant room temperature under laboratory conditions to ensure comparability of measurements. Thus, the error and nonlinearity are below ±0.05%FS, practically undetectable.

An HX711 precision 24-bit analog-to-digital converter (Avia Semiconductor Xiamen Ltd., Xiamen, China) was used to digitalize the signal from the load cell. The HX711 is specifically designed for strain gauges and other load cells. To ensure the accuracy of the force cell data, we had to calibrate the instrument. Calibration ensures that the measuring instrument is accurate and reliable. Reference weights were needed for the calibration. As the cell structure deforms during the series of measurements, it is recommended that the calibration be repeated annually. Specifications of the analog-to-digital converter: input offset drift: 0.2 mV at gain = 128 and 0.4 mV at gain = 64, input noise: 50 nV (rms) at gain = 128 rate = 0 and 90 nV (rms) at gain = 128 rate = DVDD, temperature drift: ±6 nV/°C (input offset gain = 128) and ±6 ppm/°C (gain = 128), and power supply rejection and input common mode rejection: 100 dB. The AD converter also operates at a constant temperature. To eliminate measurement errors, multiple odd-numbered measurements are taken at a single measurement time with maximum reading rates in succession. The data are stored in buffer memory and the median data are transmitted. Thus, extreme values with measurement errors are eliminated.

The key difference compared to the first-version device [33] was that the sensor had a wider range, which allowed the measurement of a broader spectrum of samples.

#### 2.1.4. Initial and Final Position

The range of motion of the traction mechanism is limited. This means that we cannot move the trolley in either direction to any arbitrary extent, as this would cause irreversible damage to our measuring instrument. To overcome this problem, limit switches were used. A limit switch (B073TYWX86, Qianxin Electronics Co., Ltd., Shanghai, China) is a switch used to limit the limit of movement of a mechanical device. The switches are mounted on the moving parts of the mechanical equipment. A limit switch is a limit position blocking block mounted on a fixed point of relative motion, or the mounting position is opposite. Unlike in the first-version device where the measurement runs until the requested number of steps are performed. That meant if the pulley system reached its endpoint before the final steps, the device would continue pulling and damage itself.

The limit switches mounted on the pulling mechanism prevent the carriage from reaching the lower limit of the mechanism and from hitting the force cell-mounted coil clamp. The limit switches are connected by an aluminum profile mounted on the trolley.

#### 2.1.5. The Microcontroller

The Arduino Mega 2560 development panel was used to process the data quantized by the HX711 sensor (Avia Semiconductor Xiamen Ltd., Xiamen, China). The Arduino Mega 2560 (RS-75-12, MEAN WELL Enterprises Co., Ltd., New Taipei City, Taiwan) is the most widely used card, based on an ATmega2560 microcontroller (Atmel Co., San Jose, CA, USA). The panel has 54 digital I/Os (Input/Output), 16 of which can be used as PWM (Pulse Width Modulation) outputs, and 16 analog inputs. The card also has a USB connector for connection to the computer, a power connector, and ICSP (In-Circuit Serial Programming) connectors. The full device is powered by a 72 W power supply module (RS-75-12, MEAN WELL Enterprises Co., Ltd., New Taipei City, Taiwan) It is ideal in terms of price/value ratio and is therefore most often used in medical–laboratory applications. The Arduino program is responsible for converting the 24-bit digital signal from the ADC to Newton values and transmitting these results in real time to the computer via the serial port. It also controls the tension motor, monitors the limit switches, controls the pump motor in the pressure measurement unit, and reads the pressure sensors. Another part of the system can also measure the pressure resistance of blood vessels.

### 2.2. The Software

For the first version of the device [33], desktop software was written in C# and the machine communicated with the computer via a USB 2.0 port. In the current version, we started to use a universal serial monitor (QtSerialMonitor 1.5 by Michal W., open-source software on GitHub Inc., San Francisco, CA, USA) where you can give commands to the instrument (start measurement, move the jaws to a specific position, set the motor speed) and export the measured data to a CSV file.

### 2.3. The Calibration

Two problems were encountered when calibrating the device: the force sensor was equipped to measure horizontal forces, and the distance between the two jaws was 15 cm, so it was not possible to calibrate with a simple standard weight. In the end, the solution was to rotate the whole device ninety degrees and place it in a water-level position. Then, test weights constructed from appropriately sized homemade coins were clamped into the jaws with a slot holder on the top to be clamped. The Arduino library for the sensor (HX711_ADC) includes a calibration program (Calibration.ino 1.2.11 by Olav K., open-source software on GitHub Inc., San Francisco, CA, USA) that helps determine the calibration constant, which you then use in your own program. After the scale had determined the value 0 in the empty state, the prepared weights were attached and weighed with a certified laboratory scale (AJCS Laborscale, Demandy, Budapest, Hungary). This value was given to the program, which then returned a constant value, which is the calibration number. This was performed 10 times per weight (*n* = 5) and then averaged and the resulting value was inserted into the Arduino kernel.

## 3. Results—Applicability

### 3.1. Test Measurements on Artificial Materials

**Figure 3:**
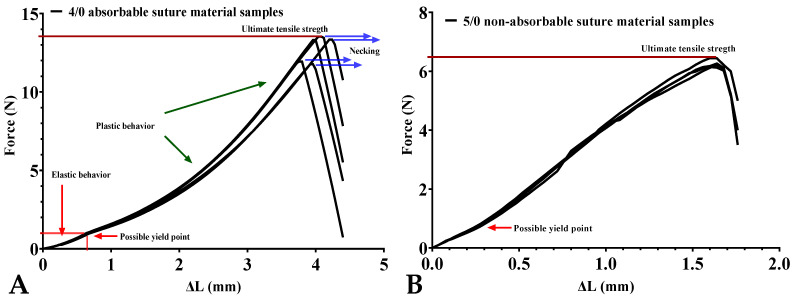
Representative superimposed tensile strength measurements stress–strain curve of different suture materials: (**A**): 4/0 absorbable polyglycolide-poly (e-caprolactone) copolymer suture material (Simfra, Kollsut, North Miami Beach, FL, USA) which we usually use for bowel anastomosis; (**B**): 5/0 non-absorbable silk suture material (Silk, SMI, Vith, Belgium) using for teaching purposes. (n = 5 per group; L_0_ = 8 mm; motor speed: 1.95 mm/s).

We carried out tests to detect any malfunctioning of the device. The tests were carried out using an absorbable and a non-absorbable suture material. We cut pieces of the same length (2 cm) and clamped them in the clamps (gauge length: distance between the HJJ-001 type grippers: L_0_ = 8 mm). Tensile strength values were calculated by mathematical derivation of the individual curves, expressing the peak values. The measurements made with these pieces of suture materials are shown in Figure 3.

The stress–strain diagrams of various materials differ in the length of the sections between each of the notable points (proportional limit, yield point, ultimate tensile strength, breaking point). In the case of ductile materials (e.g., copper, aluminum), the elastic section (L_0_-yield point) dominates. For ductile materials, such as lead or even clay, the plastic section (yield-breaking point) rule. In the case of brittle materials (glass, cast iron), the “yield point” is not present and the material fractures immediately after the elastic limit [34,35]. We observed that the curves of the measurements are almost identical in each group. The two suture materials with different compositions showed different curves. The absorbable 4/0 suture showed a characteristic of elastomers (Figure 3A), while the 5/0 non-absorbable suture resembled a kind of ductile pattern (Figure 3B, Table 1). Low variance was observed up to the yield point for both graphs. Thereafter, especially for the absorbable suture, larger variations were observed. The smaller difference between the characteristics could be due to the “noise” by HX711 ADC.

### 3.2. Test Measurements on Biopreparates

**Figure 4:**
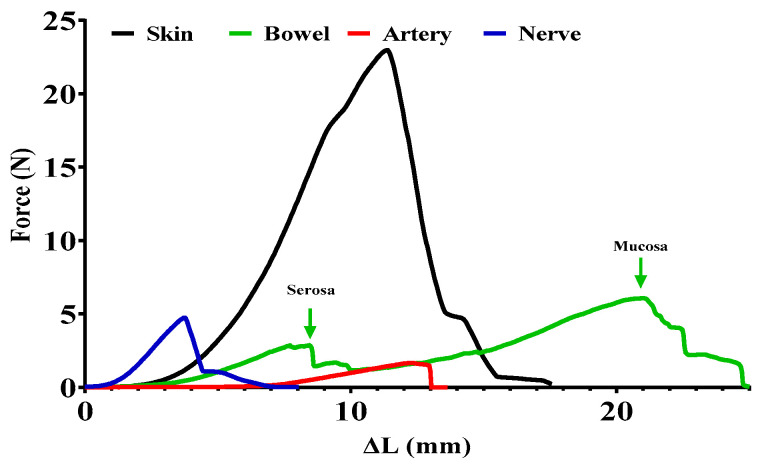
Representative tensile strength measurement stress–strain curves of different tissue biopreparates. The different mechanical properties are easily recognizable, even within a single tissue, as separate ruptures of serosa and mucosa layers (motor speed: 1.95 mm/s).

The tested tissue samples of the same width (skin, intestine: 5 mm) are always clamped at the same predetermined measuring distance (artery, nerve, skin, intestine: 8 mm) between the clamping jaws. If some tissue lesion or procedure is being examined (e.g., anastomoses made by different techniques), it is placed midway between the two jaws. The pulling force exerted by the motor (1.95 mm/s) was recorded in newtons (the gram/newton conversion (9.81 m/s^2^) was integrated into the written code of the Arduino board) as a function of elongation and exported together to a CSV file for data management. Microsoft Excel 2016 and GraphPad Prism 9.1.2 (226) software were used to analyze the ultimate tensile strength (maximum point) and slope of the stress–strain curves (Figure 4).

### 3.3. Analyzing Force–Elongation (Stress–Strain) Curves

**Figure 5:**
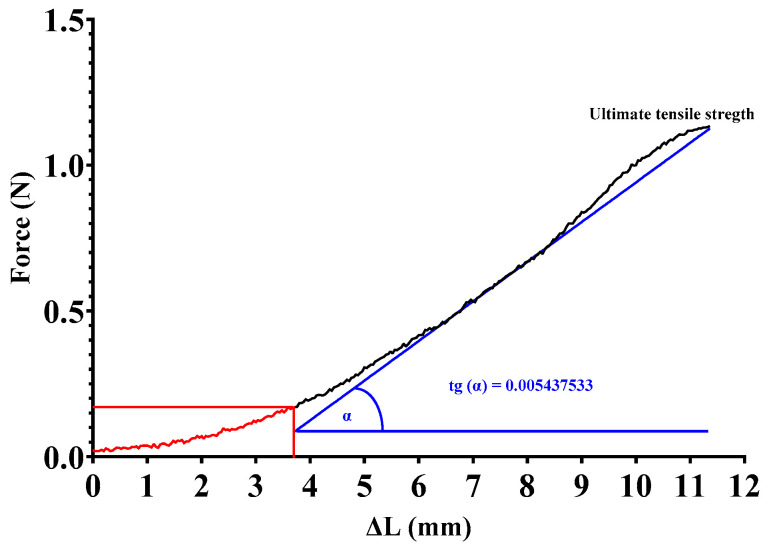
Analysis of force–elongation (stress–strain) curve. The initial part of the curve (red) was not included in the slope calculation due to its irregularity (femoral artery biopreparate of a rat; L_0_ = 8 mm; motor speed: 1.95 mm/s).

**Figure 6:**
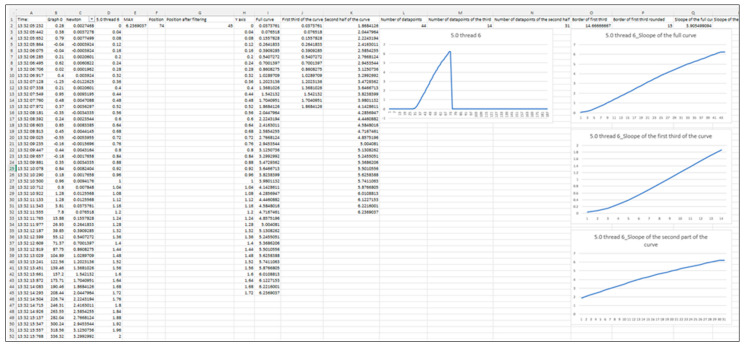
Representative analysis of the exported data. There were some irregularities in the beginning (even some negative values) so after the gram/newton conversion we applied a filter (0.0196–maximum). The entire filtered curves were divided into two parts: the first-third (0–33%) and the remaining two-thirds (34–100%). The slope of these curves was determined using the following formula in Excel = SLOPE(known_y’s, known_x’s) where x = LΔ and y = applied force. The calculation was equal to the slope of the regression line: slope = tgα = $\frac{\sum \left(x-\bar{x}\right)*\left(y-\bar{y}\right)}{\sum {\left(x-\bar{x}\right)}^{2}}$. (5/0 non-absorbable silk suture material (Silk, SMI, Belgium); L_0_ = 8 mm; motor speed: 1.95 mm/s.)

The analysis was performed on the range of curves above 0.0196 N, which, according to the accuracy of the instrument, no longer contained any irregular parts. Since the curves had a similar rupture pattern to elastomers, the initial more irregular one-third was ignored, and only the slope of the ascending section (33–100% of the whole tensile strength curve) was analyzed (tgα), which provides information on the integrity and elasticity of the collagen that primarily provides mechanical stability to the different tissues [32] (Figure 5 and Figure 6).

### 3.4. Application to Anastomosis Regeneration Studies

The device was applied in ongoing studies investigating regeneration of intestinal end-to-end and end-to-side anastomosis techniques. Table 2 shows representative data derived from our studies on vascular anastomosis (rat femoral artery) [32], small intestine anastomosis (porcine jejunum) [36], and nerve anastomosis (rat sciatic) [37] research. The data (n = 5/each) are expressed in ΔF/ΔL of a stable near-linear part of the force–elongation curves.

## 4. Discussion and Conclusions

Using the first and second versions of this device, we successfully used the measurement method to compare biomechanical properties of microvascular anastomoses using various suture types [29], and to analyze the regeneration process of end-to-end and end-to-side anastomoses [32]. According to the test measurements and experiences from research studies, the device is capable of separately detecting the tensile strength properties of various layers of the specimen (vessels, nerves, intestines) [19,27,36,37].

Biomechanical properties of biological tissues and structures have been widely studied, including blood vessels. Numerous features and parameters are known to be tested with various and diverse investigative methods, such as compression test, tensile test, tensile stress–relaxation test, creep test, dynamic mechanical analysis, burst pressure test, and compliance test, all describing a variety of parameters [21,38,39,40,41]. As mentioned earlier, there are many advantages to measuring tensile strength, and if clinical utility is considered, an example is in reconstructive surgery, where skin edges or even blood vessels are just long enough to form a suture line and therefore suture lines can tear easily if the area was moved [42,43]. The steepness of the curves may indicate the elasticity of the materials, as steeper curves are more rigid, while flatter curves may indicate a greater elasticity or even maturation problems. By analyzing the curves together with histology and microcirculation (e.g., incident dark field—IDF, or indocyanine green—ICG imaging), we can obtain more accurate information on the quality of tissue healing in research focusing on optimization and regeneration of various anastomoses [29,32]. Yield strength is the maximum stress a material can endure without experiencing permanent deformation. The yield point is the stage where the material ceases to return to its original shape and becomes irreversibly deformed. Ultimate strength represents the highest level of stress a material can endure when being stretched or pulled. Breaking strength marks the point on the stress–strain curve where the material can no longer resist the applied stress and ultimately fractures. These parameters of non-living materials are crucial in various disciplines, including mechanical engineering, materials science, and, most notably, structural engineering [34,35]. Our research is primarily concerned with the maximum tensile strength and the slope of the curves. This allows rapid conclusions to be drawn from elastomer-type curves during the healing of different tissues or even in the case of educational procedures. We believe that these two parameters are the most important once the suture has been made, since the living tissue can recover if they are not subjected to the maximum tensile strength forces compared to inanimate materials. In the future, we plan to investigate reversible/irreversible damage due to tensile forces, where we would, for example, investigate the relationship of the healing capacity with yield point.

As biological materials are not homogenous, they are not ideally elastic or plastic materials and are composed of numerous structures with various biomechanical features, there is no normal reference for these parameters. It also depends on the histomorphological features of the structures. Therefore, the necessary control measurements on normal or base values in a definitive study are always crucial.

Young’s (elastic) modulus could not be calculated, because surface data (A) and changes of A cannot be tested in this device. Therefore, we can provide only the relation of force–elongation curves data (ΔF/ΔL). An accurate, three-dimensional optical analysis of the measurement process (as samples are stretched and elongated) would be a useful further development.

The parts of the device are easily obtainable in the market, and they are very cheap. The cost of all the parts that were needed for the device was a couple of hundreds of EURs only. The force resolution is detailed enough for biopreparates/tissue samples, the accuracy is high, and the repeatability is satisfactory (Table 1). The tissue sample size can be small (for example, the diameter of the 5/0 thread is 0.1 mm); therefore, the device is feasible for experimental surgical studies, e.g., in a rat study of vascular anastomosis regeneration. The latest device can apply a force of up to 40 newtons, and could grip a 0.05–1 cm wide, 0.05–1 cm thick tissue. The length of the test piece on the rail should be between 0.3 and 5 cm The sample fixation in the device is also stable and adjustable. The device can be used in studies focusing on anastomosis techniques (on vessels, nerves, and intestines) [29,32,36,37] and related tissue regeneration.

## Figures and Tables

**Table 1:** Representative tensile strength measurement data of 5/0 non-absorbable silk suture material (Silk, SMI, Belgium) (L_0_ = 8 mm; motor speed: 1.95 mm/s).

Samples	Ultimate Tensile Strength (N)	Slope of the Full Curve	Slope of the First Third Part of the Curve	Slope of the Second Part of the Curve
1	6.145	4.149	2.862	4.246
2	6.569	4.685	6.142	4.015
3	5.847	4.307	4.381	3.941
4	6.245	4.489	4.703	4.259
5	6.331	4.073	3.429	3.839
6	6.237	3.905	3.714	3.712
7	6.404	4.976	6.222	4.356
8	5.826	4.227	3.737	3.920
9	6.178	4.4019	5.804	3.900
10	6.508	4.295	2.729	4.349
means	6.229	4.351	4.372	4.054
S.D.	0.248	0.309	1.309	0.230

**Table 2:** Representative tensile strength and force–elongation curve data (as ΔF/ΔL) of intact and anastomized rat femoral arteries, porcine jejunum, and rat sciatic nerve. Data were derived from our other relevant studies [32,36,37].

Sample Type	Group	Ultimate Tensile Strength (N)	ΔF/ΔL (N/m)
rat femoral artery	intact	1.016 ± 0.056	17.264 ± 2.771
	anastomized	0.576 ± 0.17 *	30.551 ± 19.653 *
porcine small intestine	intact	19.834 ± 5.168	198.958 ± 69.746
	anastomized	10.238 ± 2.816 *	96.285 ± 50.329 *
rat sciatic nerve	intact	17.51 ± 6.51	414.153 ± 92.867
	anastomized	1.452 ± 0.28 *	39.098 ± 13.063 *

n = 5, means ± S.D., * *p* < 0.05 vs. intact (*t*-test/Mann–Whitney RS test).

## Data Availability

The raw data supporting the conclusions of this article will be made available by the authors, without undue reservation.
